# Exosomal MATN3 of Urine-Derived Stem Cells Ameliorates Intervertebral Disc Degeneration by Antisenescence Effects and Promotes NPC Proliferation and ECM Synthesis by Activating TGF-*β*

**DOI:** 10.1155/2021/5542241

**Published:** 2021-05-27

**Authors:** Zhu Guo, WeiLiang Su, RongYao Zhou, GuoQing Zhang, Shuai Yang, XiaoLin Wu, ChenSheng Qiu, WenBin Cong, Nana Shen, JianWei Guo, Chang Liu, Shang-You Yang, DongMing Xing, Yan Wang, BoHua Chen, HongFei Xiang

**Affiliations:** ^1^Department of Orthopedics, The Affiliated Hospital of Qingdao University, Qingdao, China 266003; ^2^Department of Orthopaedics, DongChangFu People's Hospital, Liaocheng, China 252000; ^3^Department of Orthopedics, Qingdao Municipal Hospital, Qingdao, China 266011; ^4^Radiology Department, The Affiliated Hospital of Qingdao University, Qingdao, China 266003; ^5^Department of Rehabilitation, The Affiliated Hospital of Qingdao University, Qingdao, China 266003; ^6^University of Kansas, School of Medicine-Wichita, 929 N St. Francis Street, Wichita, KS, USA 67230; ^7^School of Life Sciences, Tsinghua University, Beijing, China 100084

## Abstract

**Objective:**

Low back pain (LBP) is one of the top three causes of disability in developed countries, and intervertebral disc degeneration (IDD) is a major contributor to LBP. In the process of IDD, there is a gradual decrease in nucleus pulposus cells (NPCs) and extracellular matrix (ECM). Exosomes are important exocrine mediators of stem cells that can act directly on cells for tissue repair and regeneration. In this study, we determined the antisenescence, cell proliferation promotion, and ECM modulation effects of human urine-derived stem cell (USC) exosomes (USC-exos) on degenerated intervertebral discs and explored the underlying mechanism.

**Methods and Materials:**

USCs were identified by multipotent differentiation and flow cytometry for mesenchymal stem cell- (MSC-) specific surface protein markers. USC-exos were isolated from the conditioned medium of USCs by ultracentrifugation and then analyzed by transmission electron microscopy (TEM), particle size analysis, and western blotting (WB) for exosome marker proteins. The effects of USC-exos on NPC proliferation and ECM synthesis were assessed by Cell Counting Kit-8 (CCK-8), WB, and immunofluorescence (IF) analyses. The protein differences between normal and degenerative intervertebral discs were mined, and the temporal and spatial variations in matrilin-3 (MATN3) content were determined by WB and IF in the intervertebral disc tissues. The candidate molecules that mediated the function of USC-exos were screened out and confirmed by multiple assays. Meanwhile, the mechanism underlying the candidate protein in USC-exos-induced cell proliferation and regulation of ECM synthesis promoting the activities of NPCs was explored. In addition, the effects of USC-exos on ameliorating intervertebral disc degeneration (IVD) in mice were examined by assessing computed tomography (CT), magnetic resonance imaging (MRI), and histological analyses.

**Results:**

The flow cytometry results showed that USCs were positive for CD29, CD44, and CD73, which are USC surface-specific markers, but negative for CD34 and CD45. In addition, USCs showed osteogenic, adipogenic, and chondrogenic differentiation potential. USC-exos exhibited a cup-shaped morphology, with a mean diameter of 49.7 ± 7.3 nm, and were positive for CD63 and TSG101 and negative for calnexin. USC-exos could promote NPC proliferation and ECM synthesis. The protein content of the matrilin family was significantly reduced in degenerative intervertebral discs, and the decrease in MATN3 was the most significant. USC-exos were found to be rich in MATN3 protein, and exosomal MATN3 was required for USC-exos-induced promotion of NPC proliferation and ECM synthesis, as well as alleviation of intervertebral disc degeneration in IVD rats. In addition, the effects of MATN3 in USC-exos were demonstrated to be achieved by activating TGF-*β*, which elevated the phosphorylation level of SMAD and AKT.

**Conclusions:**

Our study suggests that reduced MATN3 can be considered a characteristic of intervertebral disc degeneration. USC-exos may represent a potentially effective agent for alleviating intervertebral disc degeneration by promoting NPC proliferation and ECM synthesis by transferring the MATN3 protein.

## 1. Introduction

Low back pain (LBP) is a very common problem experienced by most people at a certain time in their life, and it is among the top three causes of disability in developed countries [1–3]. The definite causes of low back pain remain unclear; however, intervertebral disc degeneration (IDD) has been documented to be a major contributor to LBP and is the pathological basis for spinal instability, disc herniation, and other spinal degenerative diseases, which cause a considerable burden to society and families and thus are the major global public health issues [4, 5].

In disc degeneration, the main pathological change is a gradual reduction in the total NPCs and extracellular matrix (ECM). NPCs are the main functional cells responsible for ECM synthesis. The homeostatic imbalance between anabolism and catabolism leads to the loss of collagen and proteoglycan [6, 7]. Collagen type II (COL2) and proteoglycan (predominantly aggrecan (ACAN)) are crucial ECMs for discs to maintain proper function, particularly for the nucleus pulposus [8, 9]. ACAN is a biological macromolecule formed by one or more glycosaminoglycan (GAG) chains covalently connected to a core protein. It is the main noncollagen component of the intervertebral disc. The glycosaminoglycans contained in ACAN are mainly chondroitin sulfate and keratan sulfate. The rich and unique molecular characteristics of intervertebral discs allow these structures to penetrate and withstand pressure. One of the reasons for the damage and degeneration of the intervertebral disc is the degradation and loss of ACAN. COL2 is one of the most important collagen components in intervertebral discs. COL2 is the main collagen in cartilage, accounting for more than 50% of the extracellular matrix of cartilage. COL2 is mainly expressed by chondrocytes and is abundantly present in the nucleus pulposus [10, 11]. In ECM, COL2 and ACAN are the two most representative components; therefore, this experiment measured the expression of COL2 and ACAN to illustrate ECM conditions. Determining the methods of rebalancing disordered COL2 and ACAN expression and increasing their synthesis is considered a key factor for slowing down or even reversing IVD damage.

Recently, increasing evidence has revealed that mesenchymal stem cells (MSCs) can release exosomes, which are specialized extracellular vesicles that could provide therapeutic benefits [12, 13]. Exosomes are membranous vesicles with a diameter of 50-200 nm, and they contain multiple cellular components, such as proteins, nucleic acids, and lipids. Exosomes act as a cell-free mediator and transfer particular cytokines into recipient cells to achieve their therapeutic paracrine effects in inhibiting senescence, modulating metabolism, and promoting regeneration [14]. Thus, stem cell exosomes may have potential applications as effective cell-free therapeutic agents [15].

However, MSCs have a limited source and cause certain trauma to the body, which limits their application. Human urine-derived stem cells are stem cells with multidifferentiation potential obtained from human urine. These cells have a wide range of sources, are convenient to obtain, present safe and noninvasive characteristics, do not violate ethics, and represent a better choice for obtaining exosomes [16–18]. MATN3 is a member of the matrilin protein family, and it is mainly distributed in cartilage cells and plays an important role in the synthesis of cartilage ECM. Variations or decreases in its content can lead to cartilage and intervertebral disc degeneration. In previous studies, we found that USC-exos are rich in this protein.

A number of studies have shown that USC transplantation is beneficial to degenerated intervertebral discs [12, 13]. Exosomes are exocrine vesicles of stem cells that play a paracrine role and deliver a variety of biological effectors of the parent cells. Exosomes that can be absorbed by recipient cells are involved in cellular communication, signaling pathway activation, and metabolism modulation and play an important role in MSC-based therapy. Exosomes are widely distributed and readily available and have no immunogenicity; therefore, they are ideal agents for the treatment of tissue repair and regeneration [19].

In the previous study [20], the team studied the effect of USC-exos on intervertebral disc degeneration by inhibiting the apoptosis of NPCs. We now further explored the promotion of cell proliferation and ECM modulatory effects of human urine-derived stem cell (USC) exosomes (USC-exos) on degenerated intervertebral discs in cell and rat models and explored the underlying mechanism.

## 2. Materials and Methods

### 2.1. Isolation and Culture of NPCs

The experimental scheme was approved by the Ethics Committee of the Affiliated Hospital of Qingdao University (approval number: QDFY-19-012-03). Nucleus pulposus cells were obtained from 6 patients with lumbar disc herniation, and they had an average age of 30 ± 4.8. The clinical symptoms and physical examinations were consistent with the surgical indications. Before surgery, three experienced chief physicians of spine surgery and one chief physician of the imaging department evaluated their MRI, and the modified Pfirrmann scale of the lumbar intervertebral disc was grade 5. This finding indicated that the nucleus pulposus and the medial annular fiber showed low signal and the intervertebral disc was highly normal. After obtaining informed consent from the patients and their relatives and signing a donation agreement for the study, disc tissue was obtained from patients undergoing posterior lumbar foraminal surgery. The fibrous annulus (AF) and cartilaginous endplate (CEP) were carefully removed from the specimens under a microscope. After rinsing with phosphate buffered saline (PBS) 3 times, the nucleus pulposus tissue was cut to approximately 1 mm^3^ in size and then digested in 0.2% type II collagenase (Gibco, Grand Island, NY, USA) for 3 h. After digestion, a 75 *μ*m filter was used to remove tissue residue, and then, the cell suspension was centrifuged at 800 r/min for 5-10 min. Dulbecco's modified Eagle medium/F12 (DMEM/F-12) containing 10% fetal bovine serum (FBS; Gibco, Grand Island, NY, USA) and 1% penicillin-streptomycin (Gibco, Grand Island, NY, USA) was used to resuspend the NPCs; subsequently, the cells were placed in a cell incubator at 37°C with 90% N_2_, 5% CO_2_, and 5% O_2_ [21].

### 2.2. USCs Extraction

The fresh sterile urine of 6 healthy male adults (mean age, 25.5 ± 1.26) was collected under aseptic conditions. The obtained sample was centrifuged at 400 g for 10 min, the supernatant was discarded, and the cell precipitate was resuspended in phosphate buffer (PBS). The supernatant was carefully removed after centrifugation at 200 g for 10 min. The cell precipitates were resuspended in 4 ml DMEM/F-12 medium (HyClone, Utah, USA) composed of 10% FBS (Gibco, Australia), 1% penicillin-streptomycin, and RegM Singlequot growth factor additive (Lonza, Basel, Switzerland). The cells were then inoculated in 12-well plates precoated with gelatin and placed in an incubator at 37°C with 5% CO_2_. The medium was changed every two days until the cell clone was formed and then replaced with RE/MC medium for further culture. The RE cell proliferation medium was 500 ml RE cell base medium supplemented with the RegM Single kit. The MC propagation medium includes 10% FBS and 1% Glutamax added to DMEM/F-12 medium (Gibco, Japan), 1% NEAA (Gibco, Grand Island, USA), 1% Pen/Strep (Gibco, Grand Island, USA), 5 ng/ml BFGF (Peprotech, Rocky Hill, USA), 5 ng/ml PDGF-Ab (Peprotech, Rocky Hill, USA), and 5 ng/ml EGF (Peprotech, Rocky Hill, USA). Re/MC medium is a 1 : 1 mixture of Re and MC medium. When the cell density reached approximately 70%-80%, passaging was carried out, and then, P2-4 generation cells were selected for subsequent experiments.

### 2.3. Flow Cytometric Analysis of Surface Markers of USCs

After trypsin digestion, P3 USCs in a good growth state were collected and then washed with PBS 3 times after centrifugation. A cell suspension with a final concentration of 1 × 10^6^/ml was prepared. Then, 100 *μ*l of cell suspension was added to 10 *μ*l of monoclonal antibody working solution for CD29, CD34, CD44, CD45, and CD73 (Santa Cruz Biotechnology, USA) and incubated for 1 hour at room temperature in the dark. The cells were washed for another 3 times and analyzed by flow cytometry.

### 2.4. Determination of the Multidirectional Differentiation Potential of USCs

To evaluate the differentiation potential of human urine-derived stem cells, osteogenic, adipogenic, and chondrogenic differentiation was performed according to the associated kit instructions. USCs were inoculated into 6-well plates, and differentiation was induced when the cell fusion rate reached approximately 80%. Osteogenic induction differentiation medium (CYAGEN, China) was added and replaced every 3 days. After 21 days of induction, the USCs were fixed with 4% paraformaldehyde and stained with Alizarin Red for observation. The kit contained 175 ml basal medium, 20 ml serum, 2 ml penicillin-streptomycin, 2 ml glutamine, 2 ml *β*-sodium glycerophosphate, 400 *μ*l ascorbic acid, and 20 *μ*l dexamethasone. For adipogenic induction differentiation, adipogenic induction differentiation medium A (Cyagen, China) was first added and then replaced with adipogenic induction differentiation medium B (Cyagen, China) 3 days later, and after 24 h, it was replaced with liquid A again, with this process alternated 3 times. Finally, 4% paraformaldehyde was used for fixation, and oil red O staining was used for observation. Adipogenesis induction differentiation medium A contained 175 ml basal medium, 20 ml fetal bovine serum, 2 ml penicillin-streptomycin, 2 ml glutamine, 400 *μ*l insulin, 200 *μ*l 3-isobutyl-1-methyl xanthine (IBMX), 200 *μ*l dexamethasone, and 200 *μ*l rosiglitazone. The adipogenic induction differentiation medium B contained 175 ml basal medium, 20 ml fetal bovine serum, 2 ml penicillin-streptomycin, 2 ml glutamine, and 400 *μ*l insulin. During chondroblast induction, the cells were first counted, and then 2.5 × 10^5^ USCs were collected. The supernatant was discarded after centrifugation in a 15 ml centrifuge tube at 150 g for 5 min, and then, 0.5 ml chondroblast induction medium was added and changed every 3 days. Approximately 21 days later, 4% paraformaldehyde was used for fixation, paraffin embedding was followed by sectioning, and allicin blue staining was used for observation. The chondroblast induction differentiation medium kit contained 194 ml basal medium, 600 *μ*l ascorbic acid, 20 *μ*l dexamethasone, 2 ml ITS+Supplement, 200 *μ*l sodium pyruvate, 200 *μ*l proline, and 2 ml factor-*β*3 (TGF-*β*3).

### 2.5. Exosome Extraction and Identification

When the cells grew to 70-75% confluence, the culture medium was removed and washed 3 times with PBS. Serum-free medium was added, and the culture was continued for 48 h. The culture medium was then collected, centrifuged at 4°C and 500 g for 10 min to remove residual cells, and centrifuged at 4°C and 2000 g for 20 min to remove cell debris. The impurities were further removed at 4°C and 10,000 g for 30 min, and the supernatant was retained and then filtered with a 0.22 *μ*m filter membrane to remove excess particles. The supernatant was centrifuged at 4°C and 100,000 g for 2 h, and the resulting precipitate was resuspended in PBS. The exosome morphology was observed by transmission electron microscopy (TEM) (JEM-1200EX, Japan). The number and size distribution of exosomes were analyzed using a NanoSight detector (Malvern, England) and NTA detection and analysis software. USCs were cleaved to obtain their cleavage products, which were used as a negative control to conduct WB with USC-exos. Western blotting was used to detect the exosome markers CD63, TSG101, and calnexin.

### 2.6. Exosome Uptake of NPCs

P3 NPCs with good growth status were inoculated into 24-well plates for subsequent experiments after the cells adhered to the wall. First, GFP-lentivirus was transfected to observe the cell profile of NPCs. GFP virus and Lipofectamine 2000 (Thermo Fisher, Massachusetts, USA) were diluted with equal amounts of serum-free culture medium. The diluted GFP was mixed with Lipofectamine 2000 and kept at room temperature for 20 min. The mixture was added to the cell culture medium and transfected for approximately 3 h. Then, the exosomes were labeled with PKH26 (Sigma-Aldrich) fluorescent dye according to the operation instructions of the PKH26 fluorescent dye kit. The excess dye was neutralized with an equal volume of PBS containing 5% BSA. Finally, the supernatant was removed by centrifugation at 4°C for 70 min at 100,000 g and resuspended in 50 *μ*l PBS. The prepared USC-exos labeled with PKH26 were added to GFP-transfected NPCs and incubated in the dark for 12 h. After fixation with 4% paraformaldehyde for 20 min, the nuclei were stained with DAPI. The glycerin was sealed, and uptake was observed by laser confocal microscopy. The Leica Application Suite Advanced Fluorescence software was used to analyze the images in the later stage.

### 2.7. CCK-8 (Cell Counting Kit-8) Detects NPC Proliferation

NPCs were prepared into cell suspensions and counted and then seeded into 96-well plates, with 5 × 10^3^ cells in each well and each group set up with 3 duplicate wells. PBS was added to the control group, and USC-exos, USC^shMATN3^-exos, and USC^conshRNA^-exos were added to the other groups. The cell-free wells were used as blank controls. After intervention, 10 *μ*l CCK-8 solution (Meilunbio, Dalian, China) was added on days 1, 3, 5, and 7 and then cultured in a cell incubator for approximately 3 h. A microplate reader (Molecular Devices, USA) was used to detect the absorbance at 450 nm, and then, the proliferation of NPCs was calculated based on the change in absorbance.

### 2.8. *β*-Galactosidase Staining to Detect Cell Senescence

NPCs with good P3 growth were inoculated into a 6-well plate and treated according to the experimental groups after the cells adhered to the wall. After the intervention, the instructions of the *β*-galactosidase staining kit (Beyotime, China) were followed for cell senescence detection. The specific steps were as follows. Staining fixative solution was added to fix the cells at room temperature for 15 minutes. After washing 3 times with PBS, the following was added to each well: 1 ml of 930 *μ*l *β*-galactosidase staining solution C, 10 *μ*l *β*-galactosidase staining solution A, 10 *μ*l *β*-galactosidase staining *β*-galactosidase staining working solution prepared from solution B, and 50 *μ*l X-Gal solution. The cells were incubated overnight at 37°C, and the senescence of NPCs was observed under an inverted phase contrast microscope.

### 2.9. Western Blot (WB) Analysis

After the intervention, the samples were collected and then lysed in RIPA lysis buffer (Solarbio, Beijing, China) containing 1 mM phenyl methylsulfonyl fluoride (PMSF) and protease inhibitors to extract proteins. The extracted protein was tested to determine its concentration using a BCA kit (Solarbio, Beijing, China). Then, the protein and loading buffer were mixed at a ratio of 4 : 1 (*V*/*V*) and boiled for 10 minutes. The proteins were separated by sodium dodecyl sulfate-polyacrylamide gel electrophoresis (SDS-PAGE) and then transferred to a polyvinylidene fluoride (PVDF) membrane. The PVDF membrane was blocked with 5% skimmed milk powder at room temperature and then incubated overnight at 4°C with primary antibodies against CD63, TSG101, calnexin, TGF-*β*3, p-SMAD3, SMAD3, AKT, p-AKT, *β*-actin (all the above antibodies were purchased from Santa Cruz Biotechnology, USA), MATN3 (Bioss, Beijing, China), COL2 (Bioss, Beijing, China), and ACAN (Millipore, Massachusetts, USA). The membrane was incubated with a horseradish peroxidase- (HRP-) labeled secondary antibody (ABclonal, Wuhan, China) for 1 hour, and then, an ECL kit (Thermo Fisher Scientific, Rockville, MD, USA) was used for luminescence observation. The Image Lab software (Bio-Rad, Hercules, CA, USA) was used to take images and analyze them.

### 2.10. Transfection of MATN3 Lentivirus-shRNA

In the functional mechanism investigation, lentiviral shRNAs targeting MATN3 (shMATN3, sc106205V) (Santa Cruz Biotechnology, USA) and control shRNAs (Con shRNAs) were transfected before USC-exos treatment. In the feedback mechanism investigation, the overexpressed lentivirus of MATN3 (LV-MATN3) and the control lentiviral vectors (con-LV) were transfected. The lentivirus vectors were packaged by GeneChem (Shanghai, China). Transfection was conducted according to the manufacturer's instructions. Briefly, NPCs were plated into dishes 1 day before transfection. The next day, the NPCs were transfected with the lentivirus vectors at an MOI of 100 supplemented with 10 *μ*g/ml polybrene (Cyagen) for 24 h. The culture medium was replaced with fresh complete medium, and the cells were selected with 2.5 *μ*g/ml puromycin (Sigma) 72 h after transfection. Total RNA was harvested and subjected to qPCR analysis for efficiency. Forty-eight hours after transfection, the total RNA was harvested and subjected to qPCR analysis. Seventy-two hours after transfection, the total proteins were harvested and subjected to western blot analysis.

### 2.11. Quantitative Real-Time Polymerase Chain Reaction (qRT-PCR)

Total RNA was extracted from the samples using TRIzol reagent (Invitrogen, Carlsbad, CA, USA). Then, the instructions of the reverse transcription kit for qRT-PCR were followed to reverse transcription and amplify related genes. GAPDH was used as an internal reference, and each sample was set with three auxiliary holes. The primer sequences are shown in Table 1. The data obtained were analyzed using the 2^-*ΔΔ*Ct^ algorithm.

### 2.12. NPCs Immunofluorescence Test (IFT)

P3 NPCs were used for cell counting, the cell concentration was adjusted to 1 × 10^5^, and a cell climbing sheet was inserted after the cells adhered to the wall. PBS was added to the control group, 100 *μ*g/ml USC-exos was added to the USC-exos group, 100 *μ*g/ml USC^conshRNA-exos^ was added to the USC^conshRNA^-exos group, and 100 *μ*g/ml USC^shMATN3^-exos was added to the USC^shMATN3^-exos group. After 7 days of intervention, the cells were fixed with 4% paraformaldehyde for 20 minutes at room temperature and blocked with 5% BSA for 30 minutes. Primary antibodies against COL2 (Bioss, Beijing, China) and ACAN (Santa Cruz Biotechnology, USA) were added and incubated overnight at 4°C. The next day, anti-mouse (Abcam, USA) and anti-rabbit (Abclonal, USA) fluorescent secondary antibodies were added separately under dark conditions. After incubation for 1 hour at room temperature, DAPI was added. After mounting in glycerol, the cells were observed under a laser confocal microscope (Nikon, Japan) to evaluate the expression of COL2 and ACAN.

### 2.13. Rat IDD Model Establishment and Intradisc Injection

Our group purchased 20 3-month-old Sprague Dawley (SD) rats for in vivo experiments. Among them, 5 rats were regarded as the normal group without any treatment and the remaining 15 rats were regarded as the experimental group. Before the operation, the rats were anesthetized with 2% pentobarbital, and the three IVDs of each rat, namely, Co4/5, Co5/6, and Co6/7, were determined on the tail vertebrae by palpation with the aid of X-ray fluoroscopy [22]. A 20G fine needle (Hamilton, USA) was used to puncture the above three intervertebral discs, causing degeneration. Then, a 33G puncture needle (Hamilton, USA) was used to inject USC^conshRNA^-exos (100 *μ*g/ml) into Co4/5 cells, USC^shMATN3^-exos (100 *μ*g/ml) into Co5/6 cells, and PBS into Co6/7 cells (2 *μ*l each). The first injection was performed 2 weeks after the puncture and repeated 4 weeks thereafter [23], and CT (GE, USA) and 3.0 MRI (GE, USA) were performed to observe the morphological appearance of the intervertebral discs in the 4th and 8th weeks. After 8 weeks, the rats were sacrificed, and intervertebral disc samples were collected for paraffin embedding and subsequent experiments. This experimental protocol was approved by the Animal Experiment Committee of Qingdao University.

### 2.14. Hematoxylin-Eosin (HE) Staining

The samples were decalcified and fixed in formaldehyde, dehydrated, embedded in paraffin, and sectioned. Then, a hematoxylin-eosin (HE) staining kit (Solarbio, Beijing, China) was used for HE staining. Specifically, paraffin sections were deparaffinized and hydrated stained with hematoxylin staining solution for 5-20 minutes and then treated with differentiation solution for 30 seconds. After washing with warm water, the sections were placed in eosin dye solution, washed again, and soaked, and they were then dehydrated, made transparent, mounted on film, sealed with neutral gum, and observed under a microscope.

### 2.15. Safranin O-Fast Green Staining

The sample was fixed with paraformaldehyde, decalcified, dehydrated, and then embedded in paraffin. Then, the Safranin O-Fast Green Staining Kit (Solarbio, Beijing, China) was used for staining. Specifically, the sample was dewaxed in water, placed in freshly prepared Weigert's dye solution for 3-5 minutes, and then washed with water. Then, it was differentiated in acidic differentiation solution for 15 s, washed with distilled water for 10 min, dipped in the fast green staining solution for 5 min, and quickly washed with weak acid solution for 10-15 s to remove residual fast green. Then, it was placed in the Safranin O stain for 5 min, dehydrated in 95% ethanol and absolute ethanol, treated with xylene to make it transparent, and then sealed with optical resin for observations.

### 2.16. Tissue Immunofluorescence (IF) Staining

The samples were cut into frozen sections in advance for later use. Before staining, frozen sections were rewarmed at room temperature and then cleaned with TBST to remove residual optimal cutting temperature compound (OTC). After blocking with 3% BSA at room temperature for 1 hour, the MATN3 primary antibody (Bioss, Beijing, China) was added and incubated overnight at 4°C. After rewarming at room temperature for 30 minutes the next day, fluorescent secondary antibody was added, and the samples were incubated for 1 hour under dark conditions at room temperature. After DAPI was added, glycerol mount was used to observe the expression of MATN3 under a laser confocal microscope (Nikon, Japan) or stored at -20°C in the dark for subsequent observation.

### 2.17. Statistical Analysis

Each group of experiments was repeated at least three times. Continuous data are expressed as the mean ± standard deviation (SD), and nonparametric data are expressed as the median and interquartile range. One-way analysis of variance (ANOVA) was used to compare the data between groups, and the parameters of parallel groups were compared by *t*-test. *P* < 0.05 indicates that the difference is statistically significant. All data were statistically analyzed using the SPSS 20.0 software (SPSS, Chicago, IL, USA), and statistical graphs were drawn using GraphPad Prism 8 (GraphPad Software, USA).

## 3. Results

### 3.1. USC and USC-Exos Identification

**Figure 1:**
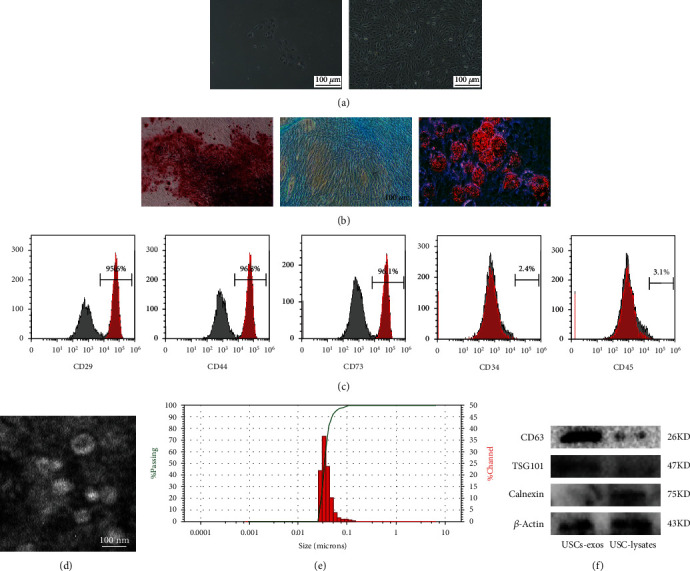
USC and USC-exos identification. (a) Morphological appearance of USCs under an optical microscope. USC colonies generally appeared at 7 to 10 days after the primary cell culture and exhibited cobblestone-like morphology under the light microscope. USCs could reach 90% confluence after 2-3 weeks of culture. (b) Multidirectional differentiation potential of USCs. After osteogenic induction, the formation of calcium nodules can be seen by staining with Alizarin Red, red-stained lipid droplets can be seen by staining with Oil Red O after the induction of fat formation, and accumulated glycosaminoglycans can be seen by staining with Alcian Blue after the induction of cartilage. (c) USC surface marker identification. USCs were positive for CD29, CD44, and CD73 but negative for CD34 and CD45. (d) TEM scanning of the USC-exos. (e) Particle size analysis of the USC-exos. (f) WB analysis of specific markers of USC-exos and USC-lysates.

USC colonies generally appeared 7 to 10 days after the primary cell culture and exhibited a cobblestone-like morphology under a light microscope. USCs had a relatively strong proliferation capacity and reached 90% confluence after 2-3 weeks of culture (Figure 1(a)). The characteristics of USCs were consistent with those described in a previous study [24].

Flow cytometry analysis showed that USCs were positive for USC surface markers CD29, CD44, and CD73 but negative for CD34 and CD45 (Figure 1(c)). USCs could differentiate into osteocytes, adipocytes, and chondrocytes when cultured in osteogenic, adipogenic, and chondrogenic conditioned culture media as previously reported [25] (Figure 1(b)). Therefore, the characteristics of USCs meet the criteria of multipotential differentiation as defined by MSCs.

USC-exos were obtained by ultrahigh-speed centrifugation. USC-exos were observed under a transmission electron microscope and showed a cup-shaped morphology with a diameter of approximately 50 nm (Figure 1(d)). The results of the particle size analysis showed that the diameter of USC-exos was 49.7 ± 7.3 nm (Figure 1(e)). WB showed that USC-exos were positive for CD63 and TSG101 but negative for calnexin (Figure 1(f)).

### 3.2. USC-Exos Resist Senescence and Promote NPC proliferation and ECM Synthesis

**Figure 2:**
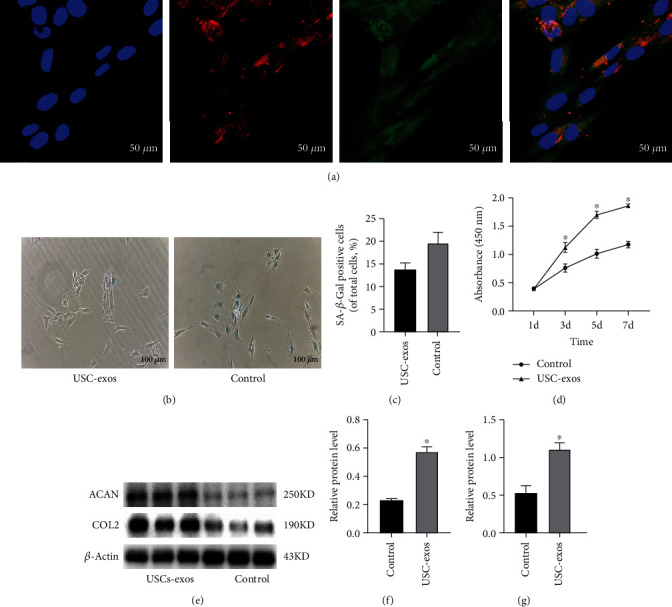
USC-exos resist senescence and promote NPC proliferation and ECM synthesis. (a) NPC uptake of USC-exos. NPCs were labeled by GFP. PKH26-labeled USC-exos were internalized to the perinuclear region of the NPCs after 3 h of incubation. (b) SA-*β*-Gal staining assay of NPCs induced by USC-exos and PBS. (c) SA-*β*-Gal staining assay showing the antisenescence effect of USC-exos. The multifield random counting method showed that the proportion of SA-*β*-Gal-positive NPCs in the USC-exos group was 13.8 ± 1.4%, which was significantly lower than that in the control group (19.6 ± 2.4%). (d) CCK-8 assay showing NPC proliferation in response to USC-exos. The absorbance at 450 nm of the USCs group was markedly higher than that of the control group at 3, 5, and 7 d (*n* = 3, ^∗^*P* < 0.05). (e–g) WB analysis for NPC ECM synthesis. The expression of ACAN and COL2 was significantly increased when NPCs were stimulated with USC-exos (each group *n* = 3, ^∗^*P* < 0.05).

To assess the effects of USC-exos on NPCs function, we first determined the NPC uptake of USC-exos. As shown in Figure 2(a), red fluorescent dye- (PKH26) labeled USC-exos were internalized into the perinuclear region of NPCs after 3 h of incubation. To determine the functional effects, USC-exos or an equal volume of PBS was added to the conditioned medium to culture NPCs for the indicated time.

A SA-*β*-Gal staining assay was utilized to examine the antisenescence effect of USC-exos, and the results showed that significantly less SA-*β*-Gal staining-positive NPCs were observed in the USC-exos group than that in the control group (Figure 2(b)). A CCK-8 analysis was performed to evaluate the effect of USC-exos on the proliferation of NPCs. The results revealed that the proliferation of NPCs was markedly promoted in response to USC-exos stimulation (Figure 2(d)).

**Figure 3:**
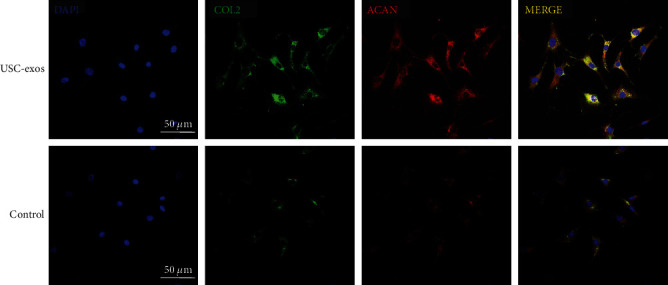
Immunofluorescence analysis of NPC ECM synthesis of COL2 and ACAN. Blue indicates DAPI, green indicates COL2, and red indicates ACAN. The expression of COL2 and ACAN was significantly increased when NPCs were induced by USC-exos.

To investigate the ECM modulation effect of USC-exos, NPCs were treated with USC-exos and PBS for 72 h. The results of western blot and immunofluorescent staining assays showed that NPCs of the USC-exos group had significantly elevated expression of COL2 and ACAN compared to the control (Figures 2(e), 2(f), and 3).

### 3.3. MATN3 Was Decreased Significantly in the Nucleus Pulposus Tissue of Intervertebral Disc

**Figure 4:**
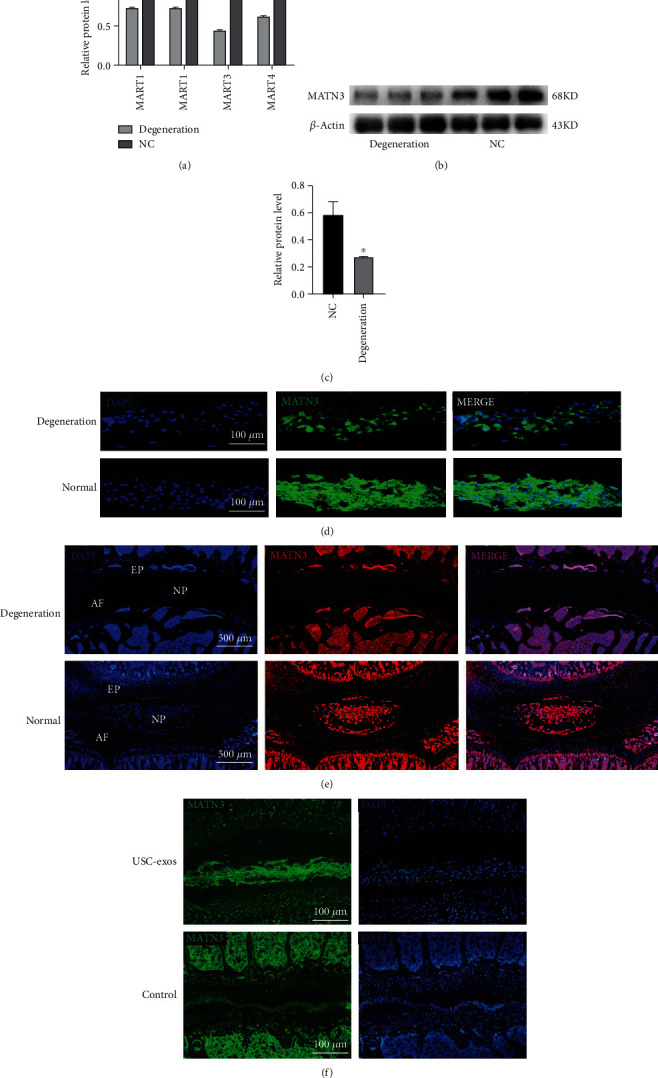
MATN3 was significantly decreased in the nucleus pulposus tissue of intervertebral discs. (a) Proteome analysis of the normal and degenerated discs of humans. The 4 matrilin family proteins were significantly decreased in the degenerated discs. MATN3 was the most differentially expressed (*n* = 5, ^∗^*P* < 0.05). (b, c) WB analysis of MATN3 expression in the normal and degenerated discs of humans. MATN3 significantly decreased in the degenerated discs (*n* = 3, ^∗^*P* < 0.05). (d) Immunofluorescence staining of human nucleus pulposus tissue for MATN3. Blue represents DAPI, and green represents MATN3. (e) Immunofluorescence image of intact rat IVDs for MATN3. (f) Exosome intervention in rat intervertebral discs was associated with the immunofluorescence results of degenerative intervertebral discs.

To investigate the potentially key proteins that lead to disc degeneration, a proteome analysis was applied in our previous study [26]. Further data mining was performed to compare the protein variation of normal and degenerated intervertebral discs, and we found that matrilin family proteins were significantly decreased in human degenerated intervertebral discs. The matrilin family has 4 members (MATN1, MATN2, MATN3, and MATN4), which are noncollagenous extracellular matrix proteins. Among them, MATN3 was the most differentially expressed (Figure 4(a)). The results of WB analysis and IF staining further confirmed the decrease in MATN3 in the degenerated human nucleus pulposus (Figures 4(b)–4(d)).

To reveal the variation of MATN3 throughout the degenerated intervertebral discs, normal and degenerated SD rat IVDs were detected for further IF staining. In normal rat IVDs, the nucleus pulposus (NP) and annulus fibrosus (AF) had a good morphological structure. MATN3 was widely distributed in the nucleus pulposus region and vertebral body and moderately distributed in the annulus fibrosus and endplate (EP). The number of MATN3-positive cells predominated in the NP and AF. However, in degenerated IVDs, the NP was unclear, and the structure of the AF was disordered. MATN3 was significantly reduced in both the nucleus pulposus and annulus fibrosus regions but not in the endplate region. Because of the partial ossification of the endplate in aged rats, there was a remarkable increase in MATN3 in endplate bone substances. However, there was no excessive expression of MATN3 in the endplates of young rats (Figure 4(e)). Immunofluorescence images of rat intervertebral discs treated with exosomes showed a significant increase in MATN3 content in the intervertebral discs, which in turn promoted extracellular matrix synthesis (Figure 4(f)).

### 3.4. Exosomal MATN3 in USCs Mediated the Antisenescence Activity and Proliferation and Promoted ECM Synthesis in NPCs

**Figure 5:**
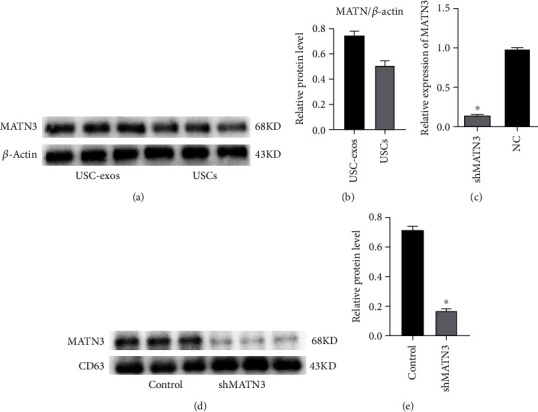
Identification of MATN3 in USC-exos and MATN3 knockdown in USCs. (a, b) WB analysis of MATN3 was rich in USC-exos (*n* = 3, ^∗^*P* < 0.05). (c) PCR analysis determined the silencing efficiency of shRNA-MATN3 to knockdown MATN3 expression in USCs (*n* = 3, ^∗^*P* < 0.05). (d, e) WB analysis of MATN3 knockdown in USC-exos (*n* = 3, ^∗^*P* < 0.05).

To investigate whether exosomal MATN3 of USCs mediates the effects, data mining was applied to previous proteomic analyses of protein expression profiles in USC-exos and their parent USCs [25]. MATN3 was found to be rich in USC-exos, and our WB results also confirmed the enrichment (Figures 5(a) and 5(b)).

MATN3 shRNA was used to knockdown the expression of MATN3 in USCs, and the inhibitory efficiency of shMATN3 was examined by qRT-PCR (Figure 5(c)). USCs transfected with shMATN3 or control shRNA (Con shRNA) were used as parental cells to generate exosomes for downstream assays. The results of WB determined the downregulation of MATN3 in exosomes from MATN3-knockdown USCs (USC^shMATN3^-exos) compared to the control exosomes from USCs transfected with Con shRNA (USC^conshRNA^-exos) (Figures 5(d) and 5(e)).

Evidence has revealed that MATN3 can directly bind to a specific integrin, which promotes the dissociation and activation of TGF-*β* by changing the conformation of the TGF-*β* precursor complex [27]. Therefore, the activation of TGF-*β* and its downstream SMAD protein and proliferation-related AKT protein [28] was further investigated.

**Figure 6:**
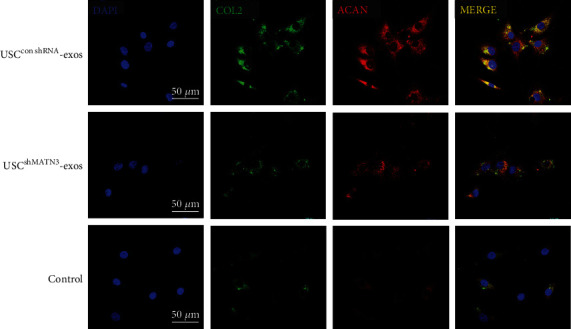
Immunofluorescence of NPC ECM synthesis for COL2 and ACAN. Blue indicates DAPI, green indicates COL2, and red indicates ACAN. The expression of COL2 and ACAN was significantly increased when NPCs were induced by USC^conshRNA^-exos; however, the promotive effects were compromised when MATN3 was knocked down in USC^shMATN3^-exos.

**Figure 7:**
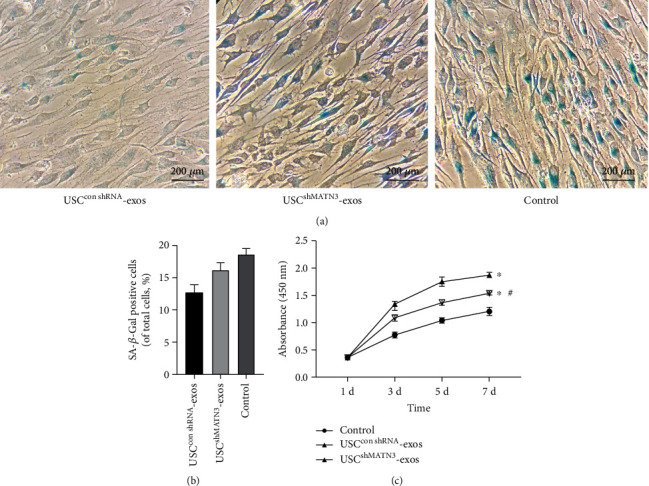
MATN3 of USC-exos mediated the antisenescence and proliferation promoting effects on NPCs. (a) SA-*β*-Gal staining assay of the NPCs induced by USC^conMATN3^-exos, USC^shMATN3^-exos, and PBS. (b) SA-*β*-Gal staining assay showing the antisenescence effect of USC-exos. Multifield random counting showed that the proportion of SA-*β*-Gal-positive NPCs in USC^conMATN3^-exos and USC^shMATN3^-exos groups was significantly lower than that in the control group. (c) CCK-8 analysis of NPCs proliferation stimulated with USC^conMATN3^-exos, USC^shMATN3^-exos, and PBS (*n* = 3 per group, ^∗^*P* < 0.05 vs. control, ^#^*P* < 0.05 vs. USC^shMATN3^-exos).

In the USC^conMATN3^-exos group, the level of TGF-*β* and the extent of p-SMAD, COL2, and ACAN expression were significantly increased. However, in the USC^shMATN3^-exos group, the promotive ability of USC-exos was markedly compromised when MATN3 expression in USC-exos was inhibited (Figure 6(a)). A SA-*β*-Gal staining assay showed that the antisenescence effect of USC-exos was mitigated, and it showed that SA-*β*-Gal staining-positive NPCs in USC^shMATN3^-exos group increased compared to those in the USC^conMATN3^-exos group but was still lower than the control group (Figure 7(a)). The CCK-8 assay also showed a decreased ability of USC-exos to promote the proliferation of NPCs when MATN3 was knocked down.

**Figure 8:**
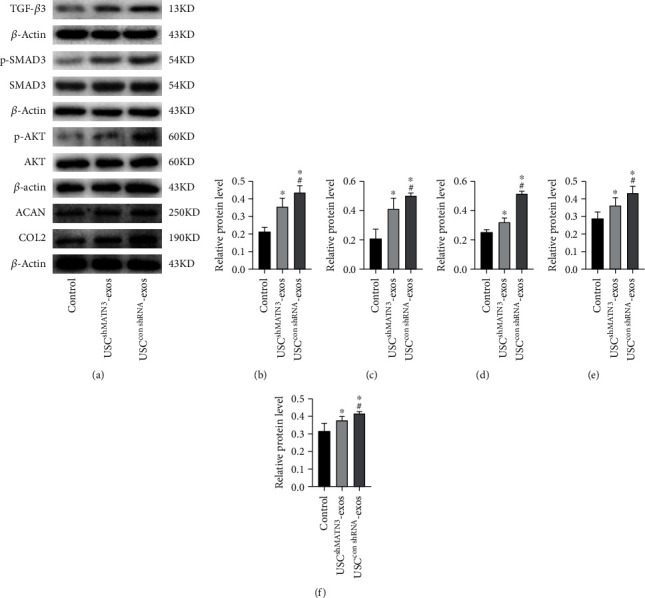
WB analysis of TGF-*β* canonical SMAD pathway and noncanonical pathway (AKT) activation (*n* = 3 per group; ^∗^*P* < 0.05 vs. control, ^#^*P* < 0.05 vs. USC^shMATN3^-exos).

In their parent USCs (Figure 7(b)), IF (Figure 8) indicated that the promotive effect of USC-exos on ECM synthesis was also suppressed once the MATN3 content was reduced in USC-exos.

Studies have reported that senescence and proliferation are associated with activating the PI3K-Akt pathway [29, 30]. Thus, we performed western blotting to detect the levels of Akt and p-Akt in NPCs following treatment with USC^shMATN3^-exos, USC^conshRNA^-exos, or an equal volume of PBS for 72 h. As shown in Figure 6(a), the ability of USC-exos to induce Akt phosphorylation was markedly compromised when MATN3 expression in USC-exos was inhibited. Collectively, our findings suggest that MATN3 is required for USC-exos-induced promotion of NPC proliferation and ECM synthesis.

## 4. Exosomal MATN3 Alleviates Intervertebral Disc Degeneration in the IVD Rat Model

**Figure 9:**
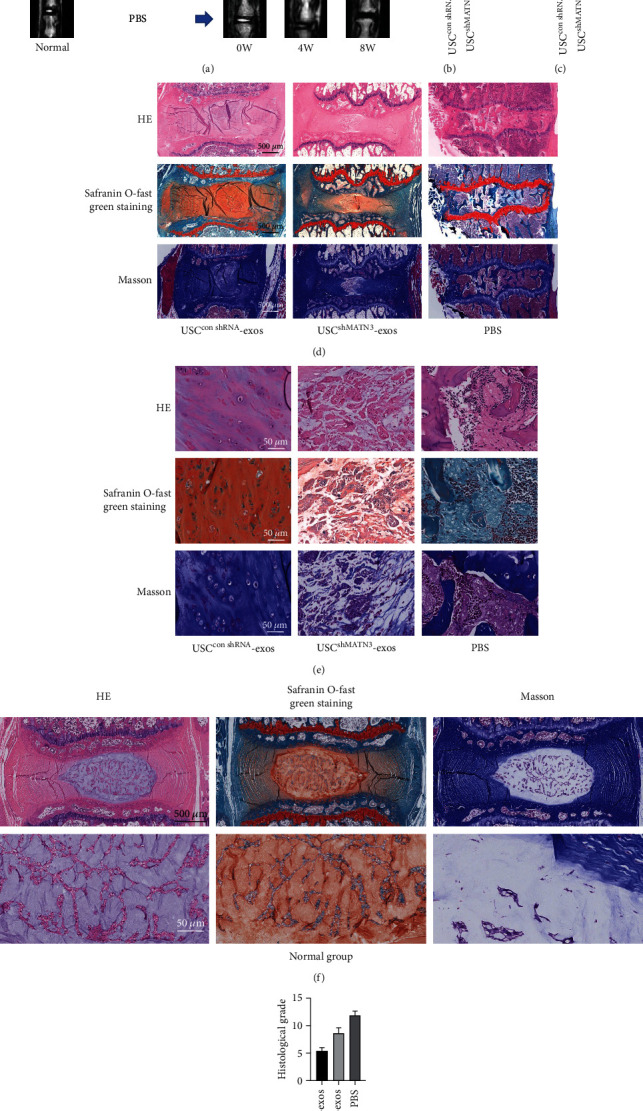
Exosomal MATN3 alleviates intervertebral disc degeneration in the IVD rat model. (a) CT and MRI images of normal rat intervertebral disc. CT and MR images of rat intervertebral discs treated with USC^conshRNA^-exos, USC^shMATN3^-exos, and PBS at 0, 4, and 8 weeks. (b, c) The change of percentage of disc height index (%DHI) was calculated based on CT measurements in each group, where (b) is the situation at 4 weeks and (c) is the situation at 8 weeks. Data are expressed as mean ± SD. ^∗^ means *P* < 0.05. (d) HE, Safranin O-fast green staining, and Masson of intact rat IVDs. (e) HE, Safranin O-fast green staining, and Masson of a partially enlarged NP of rat IVDs. (f) HE, Safranin O-fast green staining, and Masson of normal undegenerated rat intervertebral discs. (g) Histological grade was assessed by HE staining and Saffron O solid green staining. Data are expressed as mean ± SD. ^∗^ means *P* < 0.05.

To further verify the therapeutic effects of exosomal MATN3 of USC-exos, we applied USC^ConshRNA^-exos, USC^shMATN3^-exos, and an equal volume of PBS to IVD rats. The degeneration grades of rat intervertebral discs were evaluated by CT and MRI examination at 4 and 8 weeks after the intradiscal intervention (Figures 9(a) and 9(d)–9(f)). Typical disc tissue could be seen in normal, undegenerated rat intervertebral discs (Figure 9(f)). The percent disc height index (%DHI) was measured according to the results of sagittal CT reconstruction images (Figures 9(b) and 9(c)). A low %DHI indicates collapse or narrowing of the intervertebral space, which reflects the extent of degenerative changes. At 4 weeks and 8 weeks, the %DHI of the USC^conshRNA^-exos group was higher than that of the USC^conshRNA^-exos and PBS groups, and the %DHI of the USC^shMATN3^-exos group was higher than that of the PBS group. Histological grade was analyzed according to HE and Safranin O-fast green staining (Figure 9(g)).

Meanwhile, the Pfirrmann grade was based on morphological changes of the intervertebral discs in MRI, and greater degeneration corresponded to a higher grade. The Pfirrmann grade of the USC^conshRNA^-exos group was lower than that of the USC^conshRNA^-exos and PBS groups, and the Pfirrmann grade of the USC^shMATN3^-exos group was lower than that of the PBS group. In normal rats, however, there was no significant disc degeneration (Figure 9(a)).

To verify the radiographic results, further histological staining and immunohistochemical analysis were applied. As shown in Figure 9, the IVDs in the USC^conshRNA^-exos group had higher disc heights, more ECM components, and more organized NP tissues than those in the USC^shMATN3^-exos group and PBS group. However, the IVDs in the USC^shMATN3^-exos group had a lower disc height, fewer ECM components, and more disorganized NP tissues than those in the USC^conshRNA^-exos group but had a better morphological score than that of the PBS group. That is, compared to the PBS group, the intervertebral discs of the USC^conshRNA^-exos group exhibited alleviated degeneration. However, in the USC^shMATN3^-exos group, the ability to mitigate degeneration of the intervertebral disc was compromised when MATN3 was inhibited. Collectively, the radiographic results and morphological analyses indicated that full-ingredient USC-exos with MATN3 could significantly ameliorate intervertebral disc degeneration, while the beneficial effect was attenuated when MATN3 was knocked down in USC-exos.

## 5. Discussion

The main causes for IDD have not been clarified. However, a consensus has been reached that a continuous decrease in NPCs and degradation of ECM are the pathological basis of IDD [7, 31]. Therefore, finding a method of maintaining the number of NPCs and promoting the synthesis of ECM are the keys to alleviating or even reversing IDD.

In this study, we demonstrated that the content of MATN3 was significantly reduced in degenerated intervertebral discs. MATN3 is a member of the matrilin family and a noncollagenous ECM protein that shares a common structure, including the von Willebrand factor A (WFA) domain, epidermal growth factor (EGF) domain, and C-terminal coiled-coil oligomerization domain [32]. MATN3 is a cartilage-specific protein that can assemble the chondrocyte ECM. As an ECM protein, matrilin-3 can cross-link with collagen fibrils and multiple proteoglycans, playing a critical role in forming a fibrous matrix network [27]. In the past, MATN3 was found to be required for cartilage homeostasis [9]. Mutations in matrilin-3 in humans can cause many kinds of skeletal diseases, such as multiple epiphyseal dysplasia and early-onset osteoarthrosis [33]. The polymorphisms in the MATN3 gene were previously tested, and they indicated a genetic association with IDD. Mutation of the MATN3 region leads to susceptibility to spinal disc degeneration [34]. Here, for the first time, we revealed the spatial and temporal variation in MATN3 in normal and degenerated intervertebral discs. The change in MATN3 was most significant in the NP tissue and moderate in the AF. The decrease in MATN3 in the IVD could be considered a characteristic of IDD.

With the development of exosome research, an increasing number of researchers are studying exosomes as a potential treatment for intervertebral disc degeneration. Stem cell-derived exosomes may offer cell-free therapies as an alternative to traditional stem cell therapies [35]. Intervertebral disc degeneration is usually accompanied by the apoptosis of nucleus pulposus cells and the loss of extracellular matrix. The accumulation of inflammatory factors and matrix-degrading enzymes in intervertebral discs is an important reason for this phenomenon [36, 37]. In a study of degenerated and normal nucleus pulposus, Xia et al. [38] found that there were a variety of proteins related to the inflammatory response in intervertebral discs, most of which were expressed in degenerated intervertebral discs. The results showed that IL-1*β*, iNOS, COX-2, IL-6, MMP3, MMP13, and other inflammatory cytokines and extracellular matrix-degrading enzymes were significantly reduced after the addition of stem cell-derived exosomes. These results suggest that stem cell-derived exosomes can reduce the inflammatory response of intervertebral discs and the degradation of extracellular matrix. At the present stage, most experiments have used MSCs; however, this research group uniquely chose USCs. USCs not only have MSC-related characteristics but also have a number of unique advantages. USCs are a population of cells isolated from urine that have the biological properties and differentiation potential of stem cells. Although limited research has been performed on USCs, studies have confirmed that USCs can be induced to differentiate into osteoblasts, chondrocytes, smooth muscle cells, cardiomyocytes, urothelial cells, neural precursor cells, skeletal muscle cells, and adipocytes; moreover, after several generations of culture, the karyotype remains stable without tumorigenicity, the acquisition pathway is noninvasive and simple, and the culture system is stable [39–43]. Previous studies have found that the proliferation ability of stem cells is closely related to telomerase activity and telomere length. Compared with MSCs, USCs have higher telomerase activity and longer telomere sequences; therefore, they have stronger proliferation ability [44]. The above characteristics of USCs make them a better source for exosome extraction, and their application has very broad prospects.

In previous experiments, Lu et al. [45] used MSC-derived exosomes to intervene in NPCs, and the results showed that exosomes could stimulate the phenotypes of degenerated NPCs to restore undegenerated NPCs to increase the synthesis of extracellular matrix and achieve self-repair. Their studies suggest that exosomes may play a pivotal role in the endogenous repair of IVDs. In another study, Lu et al. [46] showed that MATN3 promoted the synthesis of COL2 and ACAN by promoting IL-1ra expression and inhibited the production of IL-1*β*-induced catabolic matrix proteinases, thereby delaying intervertebral disc degeneration by reducing extracellular matrix degradation. In this study, our previous experiments showed that intervention with USC-exos could reduce the degradation of extracellular matrix and promote its synthesis, which delayed the effect of intervertebral disc degeneration. The presence of MATN3 in USC-exos was verified in subsequent experiments, suggesting that USC-exos may inhibit intervertebral disc degeneration through MATN3.

Studies have shown that exosomes can promote the proliferation of NPCs and the synthesis of extracellular matrix. Moreover, exosomes are complex and contain a large number of substances, which may be released in the presence of MATN3. Our results demonstrated that USC-exos could markedly promote NPC proliferation and ECM synthesis. The promotion of USC-exos was significantly reduced after siRNA was used. At the same time, the WB method was used to identify USC-exos, and the composition of MATN3 was confirmed. By mining the data of previous proteome analyses, we found that MATN3 was rich in USC-exos, which means that USC-exos could act as a vehicle to transfer the MATN3 protein. Due to the advantages of urine-derived stem cells, the source of exosomes was optimized in this study. Moreover, the role and mechanism of MATN3 in the treatment of intervertebral disc degeneration by USC-exos were verified, with the therapeutic effect achieved by regulating the TGF-*β* content. This study provides insights for investigating exosome-based treatments for intervertebral disc degeneration.

Evidence has revealed that MATN3 can directly bind to specific integrins, which promotes the dissociation and activation of TGF-*β* by changing the conformation of the TGF-*β* precursor complex, thereby further affecting downstream gene activation [32]. Our results strongly suggested that MATN3 in USC-exos achieved the promotive function by activating TGF-*β*. TGF-*β* is a multifunctional cytokine that modulates cell fate and plasticity in a variety of tissues. The multiple cellular responses induced by TGF-*β* are mediated via the canonical SMAD pathway and noncanonical pathways, including the phosphatidylinositol 3′-kinase- (PI3K-) protein kinase B (AKT) pathway [28, 47]. TGF-*β*/SMAD pathway activation can promote the expression of COL2 and ACAN in the extracellular matrix of nucleus pulposus, and phosphorylation of AKT can promote antisenescence effects and cell proliferation [29, 30]. In the results, we demonstrated that exosomal MATN3 from USCs mediated the promotive effects. It is likely that MATN3 fulfilled its functions by activating the canonical SMAD pathway and noncanonical pathways (PI3K-AKT). We determined that MATN3 promoted the expression of TGF-*β*3 and increased the phosphorylation level of SMD and AKT NPCs. Multiple further loss-of-function assays of MATN3 suggested that exosomal MATN3 of USCs mediated the antisenescence effect and promotive effects of NPCs on proliferation and ECM synthesis. Beyond that, we verified that MATN3 in USC-exos could ameliorate IVD in the IDD rat model. The ability of USC-exos to alleviate IDD was significantly compromised when MATN3 was knocked down. The radiographic and histological analysis results indicated that the USC^conshRNA^-exos group exhibited a lower degree of IVD degeneration than the PBS group. However, in the USC^shMATN3^-exos group, the promotive effects were suppressed when MATN3 was knocked down, which indicated that MATN3 in USC-exos mediated the beneficial effects on IDD.

In addition, no significant change was observed between the PBS injection discs and the no-intervention discs, indicating a negative effect of the puncture caused by 33-gauge fine needles on intervertebral disc degeneration.

## 6. Conclusions

MATN3 is not only a noncollagenous ECM protein but also a regulator that could assist in senescence and modulate NPC proliferation and ECM homeostasis. USC-exos may be a potential therapeutic agent for IDD by transferring the MATN3 protein.

## Figures and Tables

**Table 1:** Primer sequences for quantitative real-time PCR.

Gene name	Primer sequences (5′-3′)
MATN3	Forward (F) 5′-GGTGCAGGTGTTTGCAAGAG-3′
	Reverse (R) 5′-TCCACTGTGAAGGCTTCGTC-3′
GAPDH	Forward (F) 5′-GGTATCGTGGAAGGACTC-3′
	Reverse (R) 5′-GTAGAGGCAGGGATGATG-3′

## Data Availability

The data analyzed in this research can be obtained from Zhu Guo, Yan Wang, BoHua Chen, and HongFei Xiang on reasonable request.
